# Naturally Fermented Milk From Northern Senegal: Bacterial Community Composition and Probiotic Enrichment With *Lactobacillus rhamnosus*

**DOI:** 10.3389/fmicb.2018.02218

**Published:** 2018-09-21

**Authors:** Megan Parker, Stephanie Zobrist, Chantal Donahue, Connor Edick, Kimberly Mansen, Mehdi Hassan Zade Nadjari, Margreet Heerikhuisen, Wilbert Sybesma, Douwe Molenaar, Abdoulaye Moussa Diallo, Peiman Milani, Remco Kort

**Affiliations:** ^1^PATH, Seattle, WA, United States; ^2^Microbiology and Systems Biology, Netherlands Organization for Applied Scientific Research (TNO), Amsterdam, Netherlands; ^3^Yoba for Life Foundation, Amsterdam, Netherlands; ^4^Department of Molecular Cell Biology, VU University Amsterdam, Amsterdam, Netherlands; ^5^Department of Sociology, Université Cheikh Anta Diop de Dakar, Dakar, Senegal; ^6^Helite SARL, Dakar, Senegal; ^7^ARTIS-Micropia, Amsterdam, Netherlands

**Keywords:** naturally fermented milk, *lait caillé*, bacterial community, probiotics, *Lactobacillus rhamnosus* yoba 2012, *Lactobacillus rhamnosus* GG

## Abstract

A variety of foods fermented with lactic acid bacteria serve as dietary staples in many African communities; yet, their bacterial profiles are poorly characterized. The integration of health-promoting probiotics into naturally fermented milk products could make a profound impact on human health. Here, we characterize the bacterial community composition of a naturally fermented milk product (*lait caillé*) from northern Senegal, prepared in wooden bowls (lahals) with a bacterial biofilm to steer the fermentation process. We incorporated a probiotic starter culture containing the most documented probiotic strain *Lactobacillus rhamnosus* GG (generic strain name yoba 2012) into the local fermentation process. Bar-coded 16S rRNA amplicon sequencing of *lait caillé* samples indicated that the bacterial community of *lait caillé* has high species richness with over 100 bacterial genera; however, few have high abundance. In contrast to the diverse bacterial compositions of other characterized naturally fermented milk products, the composition of *lait caillé* predominantly consists of the lactic acid bacteria *Streptococcus* and *Lactobacillus*, resembling the bacterial composition in regular yogurt. The bacterial community composition of *lait caillé* varies geographically based on the presence of some genera, including *Lactoccoccus, Enterococcus, Bifidobacterium*, and *Bacillus*, but this trend is not consistent within production communities. The diversity of bacterial communities is much higher in the *lahal* biofilm than in the naturally fermented milk products, which is in turn greater than in commercial yogurts. Addition of a starter culture with *L. rhamnosus* yoba 2012 to milk in *lahals* led to substantial growth of this probiotic bacterium during the fermentation process. Two independent quantitative PCR-analyses specific for *L. rhamnosus* yoba 2012 indicated a 20- to 60-fold increase in the total number of probiotic bacteria in the first batch after inoculation. A similar increase of the probiotic was observed in a variation of *lait caillé* prepared with carbohydrate-rich millet granules (*thiakry*) added prior to fermentation. This study shows the feasibility of integrating health-promoting probiotic strains into naturally fermented foods produced in regions with a high prevalence of malnutrition.

## Introduction

For thousands of years, fermented foods have been present in traditional diets around the world and continue to be widely consumed. Historically, communities produced fermented foods within the home, a practice that continues to this day in many settings. The ability to ferment foods enables communities to safely consume both dairy and vegetables regardless of season and to lengthen shelf life without refrigeration (Steinkraus, 1997). Furthermore, fermentation adds value to foods by enhancing their organoleptic properties, enriching nutritional properties through improved digestibility, and introducing health benefits from viable bacteria and yeasts as well as fermentation-associated modifications, including the degradation of antinutrients and toxins (Marco et al., 2017).

One of the most common traditional fermented foods is naturally fermented milk, which has a long history of consumption in Africa. In many regions of Senegal, naturally fermented milk is known as *lait caillé* and forms an integral component of the daily diet. Recent ethnographic studies have documented the role of *lait caillé* in the complementary feeding diets of infants and young children in northern Senegal (Zobrist et al., 2017, 2018). The microbial community in milk drives the fermentation process and is of great importance for the shelf life, safety, nutritional, and organoleptic properties of the final product. Naturally fermented milk offers an affordable means of delivering probiotic bacteria with specific health benefits, particularly in resource-poor settings (Franz et al., 2014). In northern Senegal, where 18% of children under five are stunted, 9% are wasted, and 18% are underweight (Agence Nationale de la Statistique et de la Démographie [ANSD], ICF, 2017), the consumption of probiotic fermented milk presents a promising opportunity to address enteric disease and malnutrition among children. We investigated the feasibility of integrating the probiotic strain *Lactobacillus rhamnosus* GG (LGG), a member of the *Lactobacillus casei* group, into the natural fermentation process of *lait caillé*.

The LGG strain has been used as a probiotic in over 30 countries and has developed into a paradigm probiotic for research on host-microbe interactions (de Vos, 2011). Its health benefits are well-documented and include the prevention and treatment of gastro-intestinal disorders including diarrhea, and stimulation of immune responses that promote vaccination or prevent allergies, among other benefits (Segers and Lebeer, 2014). In addition, the probiotic *L. rhamnosus* GG has been shown to bind and neutralize toxins known to contaminate foods, leading to a reduction of their uptake in the gastro-intestinal tract (Lahtinen et al., 2004). Further benefits of LGG include the prevention of spoilage, the enhancement of food safety, and increases in nutritional value by the delivery of vitamins (Kort et al., 2015). Previous studies have shown that inclusion of LGG in the fermentation process prevented the outgrowth of the food pathogen *Cronobacter sakazakii* in a sorghum matrix and efficiently suppressed five food pathogens in an African dairy product (Mpofu et al., 2015). Sensory evaluations of LGG-fermented milk in East Africa indicated that the properties of the final product, including texture, viscosity, creaminess, taste, sourness, sweetness and mouthfeel, were well-accepted by local communities (Kort et al., 2015).

Over the last decade, we have introduced the probiotic LGG strain to the African continent under its generic name *L. rhamnosus* yoba 2012 through the use of a novel dried starter culture (Kort and Sybesma, 2012; Sybesma et al., 2013; Kort et al., 2015). This initiative has led to access to this probiotic bacterium for 250,000 people through local production, distribution, and sale of probiotic yogurt in East Africa (Westerik et al., 2018).

In this study, we examined the bacterial community compositions in *lait caillé* from six different towns and villages in northern Senegal and explored how production process and geographic location affect the microbial communities. We analyzed the bacterial communities of *lait caillé* samples prepared in plastic buckets within small shops and in traditional wooden bowls (*lahals*) within households. Next, we compared the composition and diversity of these *lait caillé* communities with those present in purchased commercial yogurts from the same region and within the *lahal* biofilms; the *lahal* biofilms served as the starter culture for the milk naturally fermented within household wooden *lahals*. Here, we used this traditional fermentation process in northern Senegal and show for the first time that probiotic *L. rhamnosus* propagated well in naturally fermented milk. This study paves the way for integration of health-promoting strains into naturally fermented foods.

## Materials and Methods

### Sampling of Naturally Fermented Milk

**FIGURE 1:**
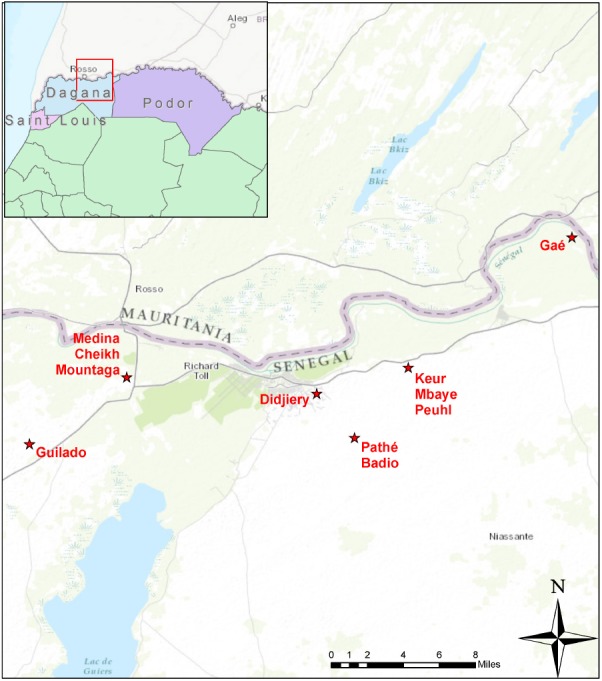
Geographic position of project sites in northern Senegal. The sites were close to the town of Richard Toll, mostly along the south bank of the river Senegal marking the border between Senegal and Mauritania. The sites include the communities of Guilado, Medina Cheikh Mountaga, Didjiery, Pathé Badio, Keur Mbaye Peuhl, and Gaé. From each of these sites, samples of *lait caillé* were collected from wooden bowls (*lahals*) from households, except for the town of Gaé, where *lait caillé* samples were collected from plastic buckets in boutiques.

**FIGURE 2:**
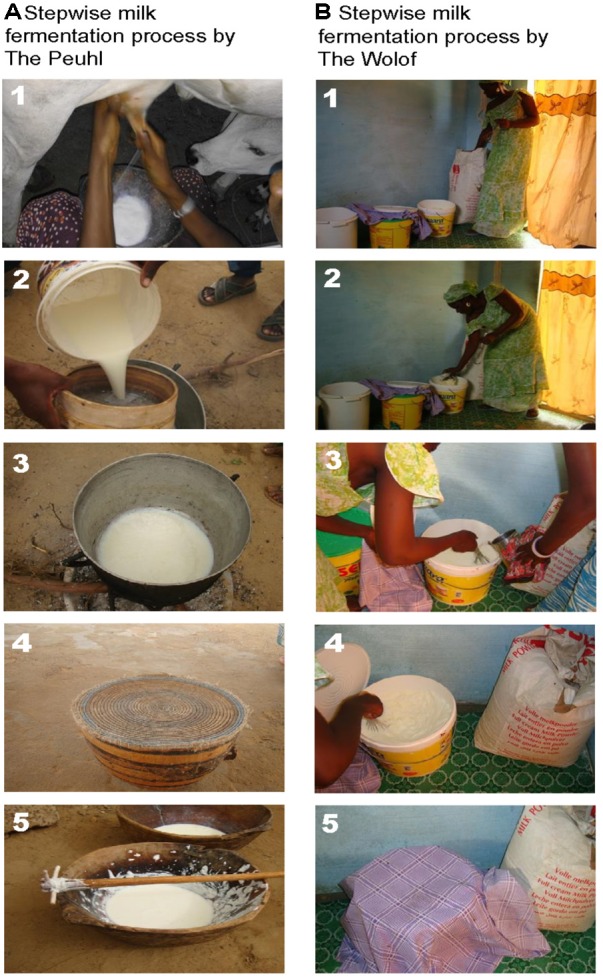
Stepwise production process of traditional fermented milk in northern Senegal. **(A)** Production process of *lait caillé* with cow’s milk in a *lahal* as practiced in households in Didjiery, Keur Mbaye Peuhl, Pathé Badio, Guilado, and Medina Cheikh Mountaga: (1) collection of milk from a cow; (2) filtration of the milk; (3) pasteurization of the milk by heating on firewood; (4) fermentation of the milk in a *lahal* for at least 12 h; (5) removal of fat and homogenization of the fermented milk with a wooden burgal. **(B)** *lait caillé* production process with milk powder in a plastic bucket in boutiques of the Gaé community: (1) milk powder is transferred to a plastic bucket; (2) water is added to the milk powder; (3) heated water is added to further dilute and warm the milk; (4) the milk is mixed with a small volume of *lait caillé* to start the fermentation (backslopping); (5) the bucket is covered with a towel and the milk is fermented for a period of 6–7 h. Written informed consent was obtained from the member of the Gaé community for the publication of these images.

We collected both domestically and commercially prepared *lait caillé* samples from communities in Senegal’s northern province of Saint-Louis (**Figure 1**). Domestic samples were prepared within homes in traditional wooden bowls (*lahals*) and the biofilm samples were obtained from the inside of the *lahal*’s surface. During the domestic preparation process, typically, one liter of cow’s milk is collected and heated in an aluminum pot, almost to the point of boiling. The milk is removed from the fire and allowed to cool before being transferred into one of the family’s traditional *lahals* and topped with a straw cover. Depending on the seasonal temperature, the milk is left to ferment in the *lahal* for 12–24 h (**Figure 2A**). Ninety-three samples of household *lahal lait caillé* samples were collected across five different communities (**Figure 1**): Didjiery (*n* = 10), Keur Mbaye Peuhl (*n* = 19), Guilado (*n* = 23), Medina Cheikh Mountaga (*n* = 22), and Pathé Badio (*n* = 19).

In contrast, *lait caillé* from more peri-urban settings is typically prepared and sold in small shops called boutiques. This process utilizes a milk powder product rather than fresh animal milk. The milk powder is mixed with freshly boiled water in a large plastic bucket, which is then covered and left to ferment for a period of 12 h. To enable the boutique fermentation process, backslopping is often used. This technique involves the re-use of a small amount of previously fermented *lait caillé* to act as a starter culture for the new batch (**Figure 2B**). Twenty-six boutique bucket *lait caillé* samples were collected from the town of Gaé (**Figure 1**).

### Sampling of Biofilms

In order to study biofilm compositions and their differences within a single community, *lahals* were sampled within 10 randomly selected households in Didjiery. Each biofilm sample was collected by placing a sterile swab halfway up the inside wall of the bowl and using it to draw a circle around the inside wall, spiraling down until it reached the center bottom point of the bowl. Duplicate samples were collected from each *lahal*, for a total of 20 samples. Each swab was placed into a test tube containing 500 μl of the quenching buffer solution RNA-later (ThermoFisher Scientific, Waltham, MA, United States) and shipped for DNA extraction and 16S rRNA amplicon sequencing, as further described below.

### Enrichment With *Lactobacillus rhamnosus*

Propagation of the probiotic bacterium *L. rhamnosus* yoba GG, also known as the generic strain *L. rhamnosus* 2012 (Kort and Sybesma, 2012; Sybesma et al., 2013), was studied in a total of nine *lahals* from three different households in the community of Didjiery. We inoculated the *lait caillé* with the starter culture, containing *L. rhamnosus* yoba 2012 and *S. thermophilus* C106 (Kort et al., 2015), and took samples 12 h after fermentation. As a control, the natural fermentation process was carried out for the same period without the starter culture. In order to test the effect of additional carbohydrates on the propagation of *L. rhamnosus* yoba 2012, we made use of locally prepared *thiakry* with millet granules, which were added to the milk together with the starter in the *lahal* prior to the fermentation period of 12 h. Samples were collected, shipped, and processed as described for the other *lait caillé* samples.

### DNA Extraction

Naturally fermented milk samples were directly quenched in 2 mL screw cap tubes in RNA-later (ThermoFisher Scientific) and shipped to TNO Zeist, The Netherlands. Subsequently, 10 μl of sample was transferred to a new 2 mL screw cap tube that contained 300 μl of lysis buffer (Agowa Mag Mini DNA Isolation Kit, LGC Ltd., United Kingdom), 500 μl zirconium beads (0.1 mm; BioSpec products, Bartlesville, Oklahoma, United States), and 500 μl of phenol saturated with Tris–HCl (pH 8.0; Carl Roth GmbH, Germany). Mechanical disruption of bacterial cells was done by bead beating for 2 min in a mini-beadbeater-8 cell disruptor (Merlin Bio-products, Breda, The Netherlands) at setting fast (homogenize). After bead beating, the samples were cooled on ice prior to a 10 min 10,000 rotations per minute centrifugation step. After another phenol extraction step of the aqueous phase, 300 μl of the aqueous phase was transferred to a new centrifugation tube pre-filled with 600 μl binding buffer and 10 μl magnetic beads (Agowa). After mixing, the suspension was left for 30 min to allow binding of the chromosomal DNA to the magnetic beads. After washing the beads, the DNA was eluted from the beads with 65 μl according to the Agowa Mag mini DNA extraction protocol.

### Bar-Coded Amplicon Sequencing

The amount of bacterial DNA present in the samples was determined by a quantitative polymerase chain reaction (qPCR) using primers specific for the bacterial 16S rRNA gene (Ciric et al., 2010). Bar-coded 16S rRNA amplicon sequencing of the V4 hypervariable region and data processing was performed according to methods described in detail by Zaura et al. (2017). Briefly, 16S rRNA V4 amplicon sequencing of 200 pg of DNA was carried out with primers including the Illumina adapters and a unique 8 nt sample index sequence key (Kozich et al., 2013). Amplicon libraries were pooled in equimolar amounts and purified using the Qiaquick Gel extraction kit (Qiagen). Paired-end sequencing of amplicons was conducted on the Illumina MiSeq platform (Illumina, Eindhoven, Netherlands). The sequence data was processed with Mothur version 1.36.1 (Schloss et al., 2009). Taxonomic names were assigned to all sequences using the Ribosomal Database Project (RDP) naïve Bayesian classifier (Wang et al., 2007).

### Quantitative PCR

The propagation of the probiotic *L. rhamnosus* in *lait caillé* samples was followed by qPCR using the 7,500 Fast Real-Time PCR System (Applied Biosystems) by the use of a previously reported protocol and designed primers (Sybesma et al., 2013). Briefly, primer-probe combinations al for quantitative polymerase chain reactions (qPCRs) were specifically designed for two genes of *L. rhamnosus* GG, referred to as PCR1, the *spaC* gene (LGG_00444), and PCR2, the *L. rhamnosus* GG-specific single-copy gene (LGG_00154). The TaqMan probes contained the minor groove binder (MGB) probe in combination with a non-fluorescent quencher and a reporter. The concentrations of DNA were deducted from a calibration curve made by the use of a dilution series of known concentrations of *L. rhamnosus* GG genomic DNA.

### Data Analysis

**FIGURE 3:**
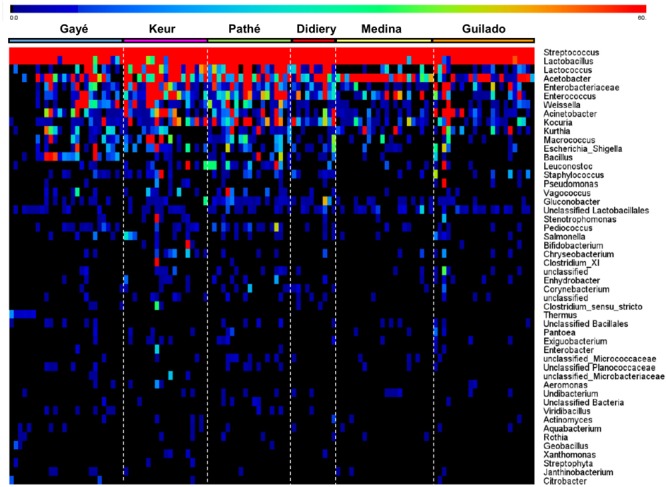
Heat map of the average top 50 bacterial genera for each community. The heat map is based on normalized 16S rRNA V4 amplicon sequence data obtained from bacterial DNA isolated from 120 *lait caillé* samples from six communities in northern Senegal. Samples were collected from plastic buckets in boutiques from Gaé (*n* = 26), and from *lahals* in households from Didjiery (*n* = 10), Keur Mbaye Peuhl (*n* = 19), Pathé Badio (*n* = 19), Guilado (*n* = 23), and Medina Cheikh Mountaga (*n* = 22). The number of observations for each bacterial genus was plotted in a rainbow scale from black to blue to green to red. Saturation was set at a value of 60 (1% of the maximum).

**FIGURE 4:**
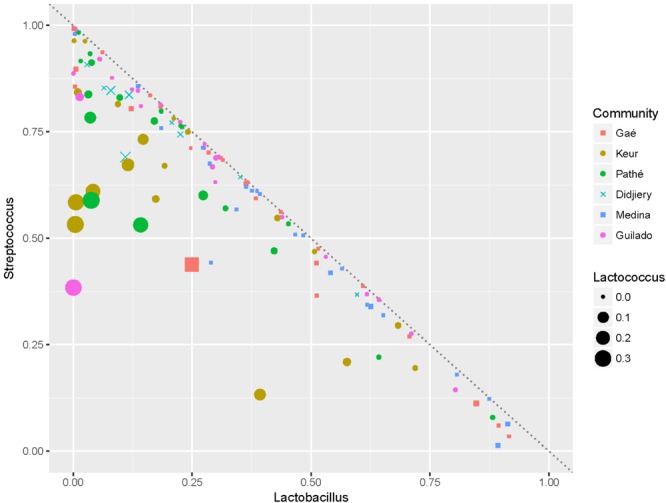
The *Streptococcus-Lactobacillus* dominance. Plot of the fractions of *Lactobacillus vs.* the fractions of *Streptococcus* in the *lait caillé* samples. The six communities are indicated by different color and symbol coding. The dotted line indicates where *f Lactobacillus* +*f Streptococcus* = 100%. The size of the symbols indicates the fraction of *Lactococcus*.

**FIGURE 5:**
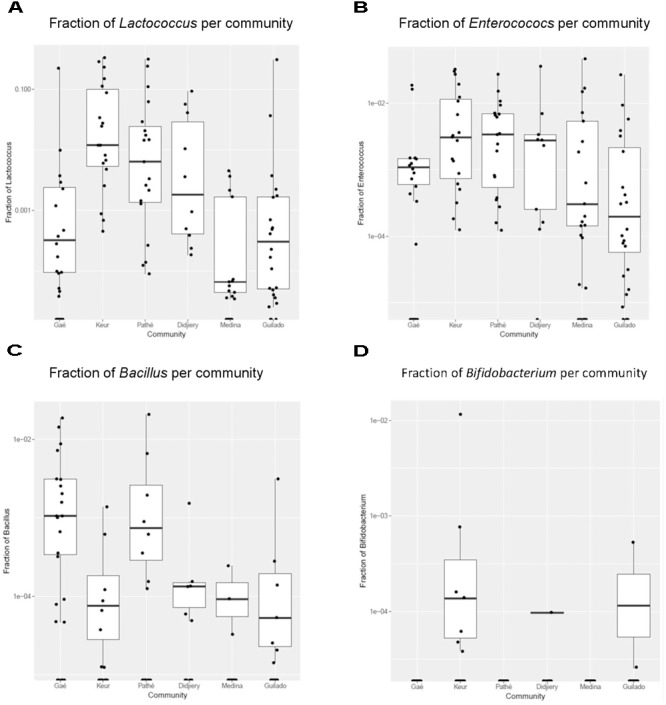
Fraction of specific genera in *lait caillé* samples per community. Box plots for genera per production community. Only genera with a *q* < 5% are shown (see **Table 1**). **(A)** fraction of *Lactococcus* per community; **(B)** fraction of *Enterococcus* per community; **(C)** fraction of *Bacillus* per community; **(D)** fraction of *Bifidobacterium* per community.

Heat maps were created using the MeV (Multi-experiment Viewer) software package version 4.9.0 (Saeed et al., 2003) to display the relative abundances of bacteria genera within and across individual samples. The heat map displays the 50 most abundant genera and is based on normalized 16S rRNA V4 amplicon sequences data obtained from *lait caillé* samples from six communities in northern Senegal (**Figure 3**). The number of observations for each bacterial genus in this heat map was plotted in a rainbow scale from black to blue to green to red. Frequency distributions of the *Streptococcus*-*Lactobacillus* dominance (**Figure 4**), and the fraction of specific genera (**Figure 5**) were calculated after exclusion of all genera that only occurred once in a sample. The Shannon indices (**Supplementary File S3**) were calculated for all samples using all identified bacterial genera.

### Availability of Data

All the 16S rRNA raw data reads of the samples sequenced in this study can be accessed under the project name ‘Bacterial Community Composition of Naturally Fermented Milk from northern Senegal’ at NCBI BioProject number PRJNA482953. The relevant datasheets are published as **Supplementary Files S1, S2**.

## Results

### Bacterial Community Composition in Naturally Fermented Milk

The composition of a total of 120 *lait caillé* samples from six communities was analyzed by bar coded amplicon sequencing. The majority of these samples (93) were collected from *lait caillé* prepared in *lahals* in households from the communities Didjiery (*n* = 10), Keur Mbaye Peuhl (*n* = 19), Guilado (*n* = 23), Medina Cheikh Mountaga (*n* = 22), and Pathé Badio (*n* = 19). A smaller number of samples was collected from *lait caillé* prepared in boutiques in plastic buckets from the town of Gaé (*n* = 26). The geographic locations of these communities have been indicated in **Figure 1**, and the stepwise preparation methods from milk to natural fermented product is shown in **Figure 2**. Overall, bacterial community compositions of all 120 samples are indicated in a heat map in **Figure 3**, with the relative abundance of the genera indicated on a color-coded scale from black to red. After exclusion of the genera that occur only once in a sample, the total number of identified genera equals 122. Among those, the 50 genera with the highest average abundance are displayed in the heat map (**Figure 3**).

Although a wide variety of genera have been identified, the *lait caillé* bacterial community is dominated by *Streptococcus* and *Lactobacillus* in all samples. These two genera constitute, on average, 61.2% and 31.5%, respectively, of the total bacterial community. This composition resembles regular yogurt, which is the product of controlled milk fermentation by two species of the same two bacterial genera. Consistent with the uncontrolled nature of its fermentation, the *lait caillé* bacterial community not only contains a wide variety of different genera, but also considerable product-to-product variation. On the higher taxonomic phylum level, the average composition comprises bacterial members of Firmicutes (96.7%), Proteobacteria (2.9%), and Actinobacteria (0.3%). Apart from *Streptococcus* and *Lactobacillus*, the Firmicutes are represented by a variety of other common lactic acid bacteria, including *Lactococcus* (2.5%), *Weisella* (0.4%), *Enterococcus* (0.4%), *Leuconostoc* (0.06%), *Vagococcus* (0.03%), and *Pediococcus* (0.02%). Amongst the Proteobacteria, the acetic acid bacteria *Acetobacter* (1.6%), *Acinetobacter* (0.4%), and enterobacteria *Escherichia*/*Shigella* (0.1%) occur at relatively high abundances. The third phylum of Actinobacteria is represented by the genus *Kocuria* (0.3%), as well as the genus *Bifidobacterium*, the latter only in a small number of samples.

### *Streptococcus* and *Lactobacillus* Dominate in Naturally Fermented Milk

The *lait caillé* samples are dominated by a limited number of genera. This is evident from the maximum fractions per genus in any of the samples (**Supplementary File S3**). In decreasing order, we identified the lactic acid bacteria *Streptococcus* (maximum 99.5%; present in 100% of the samples), *Lactobacillus* (maximum 91.6%; present in 100% of the samples), *Lactococcus* (maximum 33.4%; present in 81.5% of the samples) and the acetic acid bacterium *Acetobacter* (maximum 26.6%; present in 95.0% of the samples). A noticeable genus is the lactic acid bacterium *Leuconostoc*, which occurs at a maximum fraction of only 1.2% but does occur in more than one third of the samples.

Thus, naturally fermented milk in northern Senegal seems to be the result of fermentation by two dominating bacterial genera *Streptococcus* and *Lactobacillus* (**Figure 4**). Most samples are distributed near the line where *f Lactobacillus* + *f Streptococcus* = 100% (i.e., they are fully dominated by *Streptococcus* and *Lactobacillus*). In fact, in 78% of the samples, the sum of *Lactobacillus* and *Streptococcus* fractions exceeds 90%. Furthermore, **Figure 4** indicates that all of the samples with a high fraction of *Lactococcus* (indicated by the size of the dots) have a relatively low fraction of *Lactobacillus*. It is also evident from this plot that a high dominance of *Lactobacillus* correlates negatively with the presence of high fractions of other genera than *Streptococcus*. *Streptococcus* is dominant (more than 50%) in 9 out of 10 samples from the Didjiery community. *Lactococcus* is present at a fraction lower than 1% in 77% of the samples. High numbers (more than 1%) of *Lactococcus* occur only in 11 out of 19 samples of Keur Mbaye Peuhl, in 9 out of 19 samples of Pathé Badio samples, and in 4 out of 10 Didjiery samples.

### Correlation of Genera With Production Communities

The correlation of fractions of genera in naturally fermented milk products within the six production communities was systematically investigated by applying a Kruskal-Wallis test for each genus, including the False Discovery Rate (*q*-value) calculated according to the Benjamini-Hochberg method (**Supplementary File S4**). The four genera in the table with a *q*-value of less than 0.5% are shown in box plots with the fractions of each genus plotted on a log scale (**Figure 5** and **Table 1**). Keur Mbaye Peuhl, Pathé Badio, and Didjiery have higher fractions of *Lactococcus.* All samples from these three communities consistently contain *Lactococcus*. The samples from the other production communities contain *Lactococcus* only in a fraction of the samples. In addition, we found community-to-community variation for the genera *Enteroccoccus, Bacillus*, and *Bifidobacterium*. The fraction profile per community of *Enterococcus* resembles that of *Lactococcus*, possibly resulting from similar environmental factors in these communities. The sporeforming genus *Bacillus* is present in relatively high abundance in most of the samples in Gaé, the only community where the naturally fermented milk was prepared in plastic buckets. The genus *Bifidobacterium* was identified at low abundance in a small number of samples in three communities, with the highest frequency in Keur Mbaye Peuhl.

**Table 1:** The correlation of (fractions of) genera with the six production communities was investigated by applying a Kruskal-Wallis test for each genus.

rankID	Taxon (genus)	*Kw*-test. *p*-value	*q*-value
0.2.30.1.2.6.1	*Lactococcus*	0.0000000	0.0000000
0.2.2.1.3.1.3	*Bifidobacterium*	0.0000829	0.0026409
0.2.30.1.1.2.4	*Bacillus*	0.0000642	0.0026409
0.2.30.1.2.3.2	*Enterococcus*	0.0000866	0.0026409

*The table below shows the result, including the False Discovery Rate (q-value), calculated according to the Benjamini-Hochberg method. The four genera with the lowest q-value have been listed.*

### Diversity in *Lahal* Biofilms and Fermented Milk

**FIGURE 6:**
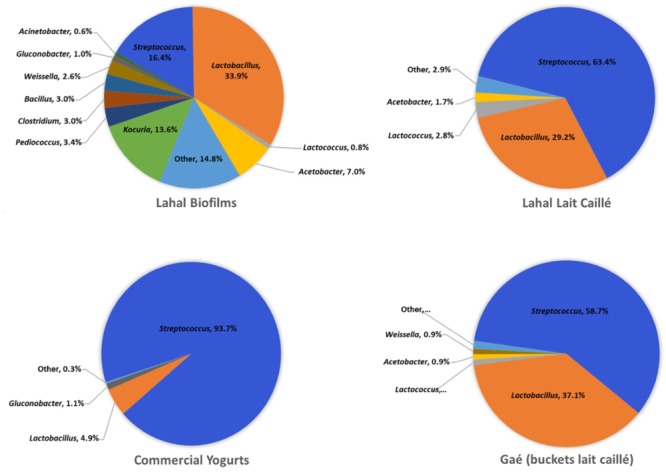
Average microbiota composition of *lahal* biofilm and fermented milk. The pie charts indicate the predominant genera (>0.5%) of household *lahal* biofilms (10 samples), household *lahal lait caillé* (93 samples from 5 communities), the boutique bucket *lait caillé* (26 samples from Gaé), and commercial yogurts (6 samples).

We compared the bacterial community composition in the *lahal* biofilm to that of the naturally fermented milk product (**Figure 6**). In contrast to the naturally fermented milk, *Streptococcus* was on average much less abundant than *Lactobacillus* in the biofilms (16 vs. 34%). However, all of the major genera identified in the naturally fermented milk were also present in the biofilm, including the lactic acid bacteria *Weisella* (2.6%), *Pediococcus* (3.4%), *Lactococcus* (0.8%), and acetic acid bacteria *Acetobacter* (7.0%) and *Gluconobacter* (1.0%). A much higher abundance was observed in the biofilm of the sporeforming bacteria *Clostridium* (3.0%) and *Bacillus* (3.0%). Most striking was the predominance in the biofilm of the actinobacterium *Kocuria* (14%), a genus that occurs in 87% of the *lait caillé* samples, although at low abundance (**Supplementary File S5**). Overall, the biofilm shows a much more even distribution of genera than the naturally fermented milk products (**Figure 6**). A further comparison of bacterial community compositions between the samples analyzed in this study indicates that the diversity in naturally fermented milk from wooden bowls is not significantly different from milk prepared in plastic buckets in shops, while commercial yogurt shows a much lower diversity. As expected, the latter bacterial community composition consists only of *Lactobacillus* and *Streptococcus*. These observed compositional differences are also evident from the average Shannon diversity indices (**Supplementary File S6**). Apart from the fermentation starters – the biofilms for the *lahals* and the backslopping method commonly used for the *lait caillé* production in buckets – some shop keepers use starters in the form of tablets for the inoculation of their milk fermentation, which are available in the local market. We also evaluated the bacterial composition in these tablets (**Supplementary File S5**) and identified that, apart from *Streptoccoccus* and *Lactobacillus*, high levels of other bacterial genera were present, including *Geobacillus* and *Anoxybacillus*, thermophilic sporeformers, which are often encountered as contaminants in milk processing environments (Zhao et al., 2013).

### Propagation of Probiotic *Lactobacillus rhamnosus* in *lait caillé*

We investigated the enrichment of *lait caillé* with the probiotic bacterium *L. rhamnosus* yoba GG, with the generic name *L. rhamnosus* yoba 2012 (Kort and Sybesma, 2012). We added the dried probiotic starter culture containing *L. rhamnosus* yoba 2012 and *S. thermophilus* C106 (Kort et al., 2015) and took samples immediately after adding the starter culture alone and after adding the starter culture with millet granules (a local food known as *thiakry*) as an additional carbohydrate source. We took a second sample after a 12 h period of fermentation. In our negative control experiment (no starter culture added), we observed no amplified product (or only a minor amount below 5 fg/μl × 10^2^ fg/μl) in the qPCR experiment with both primer combinations. Upon addition of the dried starter culture, we systematically observed higher levels at the start and a consistent 30- to 60-fold increase in the probiotic strain after the 12 h fermentation process (**Table 2**). The ratio of amplified products from PCR1/PCR2 is in all cases close to one, indicative for the presence of the *spaC* gene in the majority of the LGG bacteria in the fermented milk. The presence of the *spaC* gene confirms the genome stability as this gene is lost at relatively high frequencies upon propagation in milk affecting the probiotic properties of the strain (Sybesma et al., 2013). There appears to be no significant effect on the average growth of the probiotic bacterium when carbohydrates in the form of *thiakry* millet granules are added prior to the fermentation process (**Table 2**). The PCR reactions with both primer combinations, used for PCR1 and PCR2 for *L. rhamnosus*, provide very similar results. Together, the experiments provide clear evidence for the propagation of *L. rhamnosus* in the background of the natural milk fermentation process.

**Table 2:** Quantitative PCR showing propagation of *Lactobacillus rhamnosus* 2012 in *lait caillé* (factor of 30–60 times increase in DNA content per volume unit over a period of 12 h).

			*t* = 0 h		*t* = 12 h		*t* = 0 h	*t* = 12 h	*t* = 12 h/*t* = 0 h	*t* = 12 h/*t* = 0 h
*Lait caillé*	*Lahal*^#^	hh. ^#^	PCRl (fg/μl)	PCR2 (fg/μl)	PCRl (fg/μl)	PCR2 (fg/μl)	qPCRl/qPCR2	qPCRl/qPCR2	qPCRl	qpCR2
Control	1	1	0	2.25E + 02	9.46E + 01	1.11E + 02	NA	0.9	NA	0.5
	2	2	0	5.02E + 02	1.24E + 02	1.89E + 02	NA	0.7	NA	0.4
	3	3	1.15E + 02	NA	1.77E + 02	NA	NA	NA	1.5	NA
Probiotic *L. rhamnosus* yoba 2012	4	1	7.71E + 03	8.79E + 03	2.67E + 05	2.96E + 05	0.9	0.9	35	34
	5	2	1.07E + 04	1.47E + 04	5.07E + 05	5.53E + 05	0.7	0.9	47	38
	6	3	1.44E + 03	2.08E + 03	2.78E + 04	3.33E + 04	0.7	0.8	19	16
Probiotic *L. rhamnosus* yoba 2012 and thiakri	7	1	7.74E + 03	8.91E + 03	4.93E + 05	5.32E + 05	0.9	0.9	64	60
	8	2	7.74E + 03	9.85E + 03	2.58E + 05	2.77E + 05	0.8	0.9	33	28
	9	3	1.97E + 04	2.41E + 04	6.22E + 05	6.52E + 05	0.8	1.0	32	27

*hh. ^#^household number; PCR1, quantitative PCR with primers matching the L. rhamnosus spaC gene; PCR2, quantitative PCR with L. rhamnosus-specific primers; NA, not available as a result of sample contamination.*

## Discussion

This work illustrates that the bacterial communities of over 100 domestic samples of naturally fermented milk in northern Senegal (*lait caillé*) are without exception highly dominated by the genera *Lactobacillus* and *Streptococcus*. Studies on the microbial community compositions of naturally fermented milk products – including mabisi from Zambia (Schoustra et al., 2013), dahi from India (Shangpliang et al., 2018), and a number of others (Jans et al., 2017) – also indicate community compositions that include *Lactobacillus* and *Streptococcus*. But, in most of those cases, these two lactic acid bacteria do not dominate.

We considered a number of factors that are important in shaping the naturally fermented milk bacterial community, including milk composition, bacterial composition of the inoculum, the *lahal* biofilm, and abiotic factors. As we can discriminate lactic fermentations between the “thermophilic” and the mesophilic type, the relatively high temperatures present during the fermentation process are likely to be the main factor allowing *Streptococcus* and *Lactobacillus* to dominate over the other genera present (**Figure 3**). Indeed, the optimal growth temperatures for *S. thermophilus* and *L. delbrueckii* subsp. *bulgaricus* are 44° and 43°C, respectively, while the optimal growth temperature range for mesophilic *Lactococcus lactis* strains is much lower, between 27° and 33°C (Adamberg et al., 2003). Our temperature probe investigations into the *lahal* fermentation process confirmed exceptionally high temperatures that did not drop below 30°C within the first 14 h after adding pasteurized milk (only cooled to 43°C) (**Supplementary File S7**). Our explanation is further strengthened by the compositions of bacterial communities in naturally fermented milk in *lahals* sampled 12 h after inoculation, which almost exclusively consisted of the genera *Streptococcus* and *Lactobacillus*, as further discussed below.

Although our next generation sequencing methodology of the 16S rRNA V4 amplicon lacks the resolution to identify most bacterial species unambiguously, we identified the most abundant species in *lait caillé* by comparing the corresponding 16S rRNA V4 amplicon sequences of *Lactobacillus* and *Streptococcus* (**Supplementary File S2**) with the Basic Local Alignment Search Tool (BLAST) against the 16S rRNA database for Bacteria and Archaea. The most abundant sequences exactly matched those of the two well-known yogurt species *L. delbrueckii* and *Streptococcus thermophilus*. Interestingly, a recent review identified *Streptococcus infantarius* as a predominant lactic acid bacterium in a number of fermented milk products from different African countries (Jans et al., 2017). However, we did not identify a match with the 16S rRNA of this species among the most abundant *Streptococcus* sequences in the bacterial compositions of *lait caillé*, which may result from the relatively high fermentation temperatures favoring growth of *S. thermophilus*. In the case of the predominant *Lactococcus* sequences, we found a perfect match with the species *Lactococcus lactis*, a mesophilic strain, well-known as the major fermenting species in buttermilk. A further limitation of our study is that fungi are not included in our composition analysis, while they are known to be involved in fungal-lactic fermentations, where lactic acid bacteria and yeasts produce alcoholic milks (kefir) and moldy milks (viili) (Tamang et al., 2016). Their presence could be substantial as yeast strains of *Kluyveromyces, Saccharomyces*, and *Candida* grow well in natural fermented milk at temperatures above 30°C (Kebede et al., 2007).

The metabolic interdependencies of *L. delbrueckii* and *Streptococcus thermophilus* during the conversion of milk into yogurt have been widely studied (Sieuwerts et al., 2008). To what extent both genera really interact or lead an independent coexistence cannot be concluded from our study, as this depends on their substrate preferences, interaction with other strains, and absolute counts of these species in the fermentations. Furthermore, the high density of *Lactobacillus* inversely correlates with the presence of high fractions of genera other than *Streptococcus*. Accordingly, samples with a high fraction of *Lactococcus* from the Keur Mbaye Peuhl and Pathé Badio communities typically have low fractions of *Lactobacillus* (**Figure 4**). Thus, arguably, *Streptococcus* either coexists with *Lactobacillus* or *Lactococcus* (possibly at fermentations at relatively lower temperatures), but co-existence of high fractions of *Lactobacillus* and *Lactococcus* does not occur.

Among the less abundant genera, we identified a number of common lactic acid bacteria, including *Lactococcus, Weisella, Enterococcus, Leuconostoc, Vagococcus*, and *Pediococcus*, which are able to grow in the naturally fermented milk after extended periods of incubation at temperatures below 30°C. In addition, we identified the acetic acid forming-bacteria *Acetobacter* and *Gluconobacter* in many *lait caillé* samples. These genera probably play a role in the formation of acetic acid from ethanol produced by yeasts. In addition, a number of enterobacteria were identified in the samples, including *Escherichia*/*Shigella, Salmonella*, and *Enterococcus.* These genera do not necessarily pose a food safety concern as they are commonly identified in natural fermented milk and mostly non-pathogenic (Shangpliang et al., 2018). One of the most remarkable genera that has been frequently identified, particularly in the Keur Mbaye Peuhl and Pathé Badio communities, is the actinobacterium *Kocuria*. Although this genus has been identified previously in the non-fermented milk of ruminants (Li et al., 2017), it is tempting to speculate that its presence in the naturally fermented milk products is related to inoculation from the *lahal* biofilms, as these biofilms contain relatively large amounts of the genus *Kocuria* (14% on average, **Supplementary File S2**).

We investigated if there were community-dependent bacterial populations among the seven communities we studied (**Figure 5**). In agreement with previous studies (Schoustra et al., 2013), we found very little evidence for specific communities on the basis of geographical distribution. The picture that emerges from the boxplots in **Figure 5** is that the variation of the occurrence of genera is high, both between and within production communities. Some genera do occur at higher fractions in some samples, but not always consistently within one production community. Apart from the more frequent presence of *Lactococcus* and *Enterococcus* at relatively high abundance in the samples, in three of the six communities, there is no clear evidence of typical microbial composition by region. The *Lactococcus* and *Enterococcus* genera are typically involved in milk fermentations. *Lactococcus* is known to ferment milk into dairy products other than yogurt, such as cheese, sour cream, and buttermilk (Marco et al., 2017). Although the presence of *Enterococcus* has been considered as an indication of insufficient hygiene during milk processing, the genus also contains a number of probiotic species and is known to be involved in flavor development during the process of milk fermentation, possibly resulting in a specific taste in *Enterococcus*-containing fermented milk products (Giraffa, 2003).

To a large extent, the natural inoculum (starter) will control the bacterial composition of the *lait caillé* end product, as it obviously provides the source of bacteria, of which only a limited amount will be able to propagate in the milk as a result of the specific environmental conditions. Indeed, all of the major bacterial genera we identified in the *lahal* biofilm were present in the *lait caillé*, albeit at different abundances (compare **Figures 3, 6**). The biofilm, however, is much more diverse, as is evident from the Shannon diversity indices (**Supplementary File S6**) and contains on average higher amounts of *Lactobacillus* (34%) than *Streptoccocus* (16%). Strikingly, on average it also contains higher amounts of spore-forming bacteria of the genera *Bacillus* and *Clostridium*, which may be able to persist in the biofilm.

Finally, we tested the feasibility of integrating probiotic health features into *lait caillé*. To do this, we incorporated a dried probiotic starter culture containing *L. rhamnosus* yoba 2012 and *S. thermophilus* C106 into the local fermentation process (Kort et al., 2015). The results indicated that the probiotic was able to propagate in the rich bacterial environment within the *lahals*. Since the probiotic *L. rhamnosus* yoba 2012 is not capable of independent growth in milk, we assume that growth of the probiotic is efficient in the *lait caillé* as a result of sufficient activity of proteolytic strains, including the *S. thermophilus* C106 strain present in the starter culture (Kort et al., 2015).

We collected and analyzed microbiota data to evaluate effects of the addition of *L. rhamnosus* yoba 2012 on microbial populations in *lait caillé* (data not shown). However, we could not draw any firm conclusions from these analyses, as the V4 amplicon sequencing data did not allow to discriminate on the species level to unambiguously separate *L. rhamnosus* from the other *Lactobacillus* species present in the sample. Furthermore, we collected the *lait caillé* samples after a relatively short period of 12 h of fermentation, while most of the other *lait caillé* products evaluated in the study, had been fermented for longer periods of time. Therefore, we almost exclusively identified the genera *Lactobacillus* and *Streptococcus*. This is in line with the notion that first the genera of *Lactobacillus* and *Streptococcus* ferment the milk and that the diversity of the bacterial community in naturally fermented milk will increase after the first 12 h of fermentation.

As our microbiota analyses did not allow us to monitor growth of the probiotic *Lactobacillus rhamnosus* yoba 2012 during the process of fermentation, we carried out quantitative PCR experiments with primers specifically directed against this probiotic bacterium. These experiments revealed relatively high levels of *L. rhamnosus* yoba 2012 in naturally fermented milk after a single round of fermentation. It remains to be seen if the probiotic strain is able to invade the *lahal* biofilms and sustain in the bacterial community during multiple rounds of fermentation. If not, a novel starter culture needs to be purchased for each fermentation, making the introduction of the *L. rhamnosus* probiotic more sustainable for the commercial applications of *lait caillé* than small-scale productions at the household level in *lahals*.

Health benefits of *L. rhamnosus* GG include the prevention and treatment of gastro-intestinal infections and diarrhea. This is highly relevant in sub-Saharan Africa, where diarrheal diseases accounted for more than 300,000 deaths of children under age five in 2015 (Troeger et al., 2017). In addition, *L. rhamnosus* GG has been shown to stimulate immune responses, bind toxins, suppress pathogens and enhance food safety. This study paves the way for the future integration of such health-promoting probiotic properties into locally fermented foods.

## Author Contributions

MP, PM, and RK designed the study, analyzed the data, and drafted the manuscript. MP, AMD, PM, and RK carried out the field work and sampling in northern Senegal. DM performed data analysis and prepared **Figures 4, 5**. MHZ performed the qPCR experiments. MH carried out amplicon sequencing and data processing. WS and RK developed the probiotic starter culture. All authors read, corrected, and approved the final manuscript.

## Conflict of Interest Statement

RK and WS are the founders of the Yoba for Life foundation (2009), a non-profit organization, accredited by the Dutch Tax Authorities as a Public Benevolent Institution (PBI), which aims to promote local production and consumption of fermented products in Africa. African fermented products made with the Yoba starter culture are not marketed by the foundation as such, but the Yoba for Life foundation stimulates local production and ownership, allowing income-generating activities for African small-scale entrepreneurs in the food sector. The Yoba for Life foundation distributes and sells ready-to-use sachets with dried bacterial starter cultures at cost price, through a network of partners and volunteers to facilitate the local production of dairy and cereal-based products by controlled bacterial fermentation. The Yoba starter culture contains *L. rhamnosus* yoba 2012, which is the generic variant of *L. rhamnosus* GG. The remaining authors declare that the research was conducted in the absence of any commercial or financial relationships that could be construed as a potential conflict of interest.

## Supplementary Material

FILE S1 Raw data sheet sequence reads. The data sheet contains all the sequence reads for all taxons up to the genus level identified in the naturally fermented milk samples of all six communities, collected starter cultures, commercial yogurts, and *lahal* biofilms.

FILE S2 Raw data sheet unique sequences. The data sheet contains all the unique 16S rRNA V4 sequences for all taxons up to the genus level identified in the naturally fermented milk samples of all six communities, collected starter cultures, commercial yogurts, and *lahal* biofilms.

FILE S3 Maximum fraction, fraction of samples, average fraction, and median fraction for each genus.

FILE S4 Kruskal-Wallis test for the correlation of each genus in naturally fermented milk products and the six production communities, including the False Discovery Rate (*q*-value) calculated according to the Benjamini-Hochberg method.

FILE S5 Heat maps of bacterial community compositions of starters tablets (10) and *lahal* biofilms (20).

FILE S6 The range of Shannon Indices (H’) across the samples of biofilms, household *lait caillé*, boutique bucket *lait caillé*, and commercial yogurts.

FILE S7 Average temperature profiles of overnight incubations of household *lait caillé* fermentations of three different *lahals* recorded in the community of Didjiery.

## References

[B1] Adamberg K. Kask S. Laht T. M. Paalme T. (2003). The effect of temperature and pH on the growth of lactic acid bacteria: a pH-auxostat study. *Int. J. Food Microbiol.* 85 171–183. 10.1016/S0168-1605(02)00537-8 12810281

[B2] Agence Nationale de la Statistique et de la Démographie [ANSD], ICF (2017). *Sénégal. Enquête Démographique et de Santé Continue (EDS Continue 2016).* Rockville, MD: ANSD and ICF.

[B3] Ciric L. Pratten J. Wilson M. Spratt D. (2010). Development of a novel multi-triplex qPCR method for the assessment of bacterial community structure in oral populations. *Environ. Microbiol. Rep.* 2 770–774. 10.1111/j.1758-2229.2010.00183 23766283

[B4] de Vos W. M. (2011). Systems solutions by lactic acid bacteria: from paradigms to practice. *Microb. Cell Fact.* 10(Suppl. 1):S2. 10.1186/1475-2859-10-S1-S2 21995776 PMC3231926

[B5] Franz C. Huch M. Mathara J. M. Abriouel H. Benomar N. Reid G. (2014). African fermented foods and probiotics. *Int. J. Food Microbiol.* 190 84–96. 10.1016/j.ijfoodmicro.2014.08.033 25203619

[B6] Giraffa G. (2003). Functionality of Enterococci in dairy products. *Int. J. Food Microbiol.* 88 215–222. 10.1016/S0168-1605(03)00183-1 14596993

[B7] Jans C. Meile L. Kaindi D. W. M. Kogi-Makau W. Lamuka P. Renault P. (2017). African fermented dairy products – overview of predominant technologically important microorganisms focusing on African *Streptococcus infantarius* variants and potential future applications for enhanced food safety and security. *Int. J. Food Microbiol.* 250 27–36. 10.1016/j.ijfoodmicro.2017.03.012 28364623

[B8] Kebede A. Viljoen B. Gadaga H. Narvhus J. (2007). Survival and growth of yeasts in sethemi, South African naturally fermented milk. *Food Technol. Biotechnol.* 45 21–26.

[B9] Kort R. Sybesma W. (2012). Probiotics for every body. *Trends Biotechnol.* 30 613–615. 10.1016/j.tibtech.2012.09.002 23031355

[B10] Kort R. Westerik N. Mariela Serrano L. Douillard F. P. Gottstein W. Mukisa I. M. (2015). A novel consortium of *Lactobacillus rhamnosus* and *Streptococcus thermophilus* for increased access to functional fermented foods. *Microb. Cell Fact.* 14:195. 10.1186/s12934-015-0370-x 26643044 PMC4672519

[B11] Kozich J. J. Westcott S. L. Baxter N. T. Highlander S. K. Schloss P. D. (2013). Development of a dual-index sequencing strategy and curation pipeline for analyzing amplicon sequence data on the MiSeq illumina sequencing platform. *Appl. Environ. Microbiol.* 79 5112–5120. 10.1128/AEM.01043-13 23793624 PMC3753973

[B12] Lahtinen S. J. Haskard C. A. Ouwehand A. C. Salminen S. J. Ahokas J. T. (2004). Binding of aflatoxin B1 to cell wall components of *Lactobacillus rhamnosus* strain GG. *Food Addit. Contam.* 21 158–164. 10.1080/02652030310001639521 14754638

[B13] Li Z. Wright A. G. Yang Y. Si H. Li G. (2017). Unique bacteria community composition and co-occurrence in the milk of different ruminants. *Sci. Rep.* 7:40950. 10.1038/srep40950 28098228 PMC5241872

[B14] Marco M. L. Heeney D. Binda S. Cifelli C. J. Cotter P. D. Foligne B. (2017). Health benefits of fermented foods: microbiota and beyond. *Curr. Opin. Biotechnol.* 44 94–102. 10.1016/j.copbio.2016.11.010 27998788

[B15] Mpofu A. A. Linnemann A. R. Nout M. J. Zwietering M. H. Smid E. J. den Besten H. M. (2015). Inactivation of bacterial pathogens in yoba mutandabota, a dairy product fermented with the probiotic *Lactobacillus rhamnosus* yoba. *Int. J. Food Microbiol.* 217 42–48. 10.1016/j.ijfoodmicro.2015.09.016 26490648

[B16] Saeed A. I. Sharov V. White J. Li J. Liang W. Bhagabati N. (2003). TM4: a free, open-source system for microarray data management and analysis. *Biotechniques* 34 374–378. 12613259 10.2144/03342mt01

[B17] Schloss P. D. Westcott S. L. Ryabin T. Hall J. R. Hartmann M. Hollister E. B. (2009). Introducing mothur: open-source, platform-independent, community-supported software for describing and comparing microbial communities. *Appl. Environ. Microbiol.* 75 7537–7541. 10.1128/AEM.01541-09 19801464 PMC2786419

[B18] Schoustra S. E. Kasase C. Toarta C. Kassen R. Poulain A. J. (2013). Microbial community structure of three traditional Zambian fermented products: mabisi, chibwantu and munkoyo. *PLoS One* 8:e63948. 10.1371/journal.pone.0063948 23691123 PMC3653860

[B19] Segers M. E. Lebeer S. (2014). Towards a better understanding of *Lactobacillus rhamnosus* GG—host interactions. *Microb. Cell Fact.* 13(Suppl. 1):S7. 10.1186/1475-2859-13-S1-S7 25186587 PMC4155824

[B20] Shangpliang H. N. J. Rai R. Keisam S. Jeyaram K. Tamang J. P. (2018). Bacterial community in naturally fermented milk products of Arunachal Pradesh and Sikkim of India analysed by high-throughput amplicon sequencing. *Sci. Rep.* 8:1532. 10.1038/s41598-018-19524-6 29367606 PMC5784140

[B21] Sieuwerts S. de Bok F. A. Hugenholtz J. van Hylckama Vlieg J. E. (2008). Unraveling microbial interactions in food fermentations: from classical to genomics approaches. *Appl. Environ. Microbiol.* 74 4997–5007. 10.1128/AEM.00113-08 18567682 PMC2519258

[B22] Steinkraus K. H. (1997). Classification of fermented foods: worldwide review of household fermentation techniques. *Food Control* 8 311–317. 10.1016/S0956-7135(97)00050-9

[B23] Sybesma W. Molenaar D. van IJcken W. Venema K. Kort R. (2013). Genome instability in *Lactobacillus rhamnosus* GG. *Appl. Environ. Microbiol.* 79 2233–2239. 10.1128/AEM.03566-12 23354703 PMC3623246

[B24] Tamang J. P. Watanabe K. Holzapfel W. H. (2016). Diversity of microorganisms in global fermented foods and beverages. *Front. Microbiol.* 7:377. 10.3389/fmicb.2016.00377 27047484 PMC4805592

[B25] Troeger C. Forouzanfar M. Rao P. C. Khalil I. Brown A. Reiner R. C. (2017). Estimates of global, regional, and national morbidity, mortality, and aetiologies of diarrhoeal diseases: a systematic analysis for the global burden of disease study 2015. *Lancet Infect. Dis.* 17 909–948. 10.1016/S1473-3099(17)30276-1 28579426 PMC5589208

[B26] Wang Q. Garrity G. M. Tiedje J. M. Cole J. R. (2007). Naïve bayesian classifier for rapid assignment of rrna sequences into the new bacterial taxonomy. *Appl. Environ. Microbiol.* 73 5261–5267. 10.1128/AEM.00062-07 17586664 PMC1950982

[B27] Westerik N. Kort R. Sybesma W. Reid G. (2018). *Lactobacillus rhamnosus* probiotic food as a tool for empowerment across the value chain in Africa. *Front. Microbiol.* 9:1501. 10.3389/fmicb.2018.01501 30042747 PMC6048217

[B28] Zaura E. Brandt B. W. Prodan A. Teixeira de Mattos M. J. Imangaliyev S. Kool J. (2017). On the ecosystemic network of saliva in healthy young adults. *ISME J.* 11 1218–1231. 10.1038/ismej.2016.199 28072421 PMC5475835

[B29] Zhao Y. Caspers M. P. Metselaar K. I. de Boer P. Roeselers G. Moezelaar R. (2013). Abiotic and microbiotic factors controlling biofilm formation by thermophilic sporeformers. *Appl. Environ. Microbiol.* 79 5652–5660. 10.1128/AEM.00949-13 23851093 PMC3754176

[B30] Zobrist S. Kalra N. Pelto G. Wittenbrink B. Milani P. Diallo A. M. (2017). Results of applying cultural domain analysis techniques and implications for the design of complementary feeding interventions in northern Senegal. *Food Nutr. Bull.* 38 512–527. 10.1177/0379572117720749 29065728

[B31] Zobrist S. Kalra N. Pelto G. Wittenbrink B. Milani P. Diallo A. M. (2018). Using cognitive mapping to understand Senegalese infant and young child feeding decisions. *Matern. Child Nutr.* 14:e12542. 10.1111/mcn.12542 29110396 PMC6865947

