# Supplementary material for: Naturally Fermented Milk From Northern Senegal: Bacterial Community Composition and Probiotic Enrichment With Lactobacillus rhamnosus
Source: Front Microbiol. 2018 Sep 21;9:2218. doi: 10.3389/fmicb.2018.02218 (PMC6160551; doi:10.3389/fmicb.2018.02218)
Supplement: FILE S1 — Raw data sheet sequence reads. The data sheet contains all the sequence reads for all taxons up to the genus level identified in the naturally fermented milk samples of all six communities, collected starter cultures, commercial yogurts, and lahal biofilms. [file Data_Sheet_1.ZIP › Supplemental file S4.pdf]

Kruskal-Wallis test for the correlation of each genus in natural fermented milk products and the six production communities, including the False Discovery Rate (q-value) calculated according to the Benjamini-Hochberg method.

rankID	taxon	kwtest.pvalue	qvalue
0.2.30.1.2.6.1	Lactococcus	0.0000000	0.0000000
0.2.2.1.3.1.3	Bifidobacterium	0.0000829	0.0026409
0.2.30.1.1.2.4	Bacillus	0.0000642	0.0026409
0.2.30.1.2.3.2	Enterococcus	0.0000866	0.0026409
0.2.21.6.1.1.44	unclassified	0.0002655	0.0053993
0.2.21.6.16.2.20	Xanthomonas	0.0002441	0.0053993
0.2.21.2.8.1.13	Gluconobacter	0.0003244	0.0056532
0.2.12.1.2.1.4	Thermus	0.0004410	0.0067254
0.2.2.1.2.2.2	Actinomyces	0.0006383	0.0086520
0.2.30.1.2.5.2	Leuconostoc	0.0008378	0.0102206
0.2.30.1.2.5.4	Weissella	0.0010562	0.0117139
0.2.21.6.1.1.14	Escherichia_Shigella	0.0016020	0.0139602
0.2.30.1.1.12.17	unclassified	0.0015966	0.0139602
0.2.36.1.1.1.1	unclassified	0.0015757	0.0139602
0.2.2.1.2.25.5	Kocuria	0.0020865	0.0169704
0.2.2.1.2.24.35	unclassified	0.0033097	0.0240874
0.2.30.1.2.3.6	Vagococcus	0.0033564	0.0240874
0.2.21.2.8.1.1	Acetobacter	0.0037387	0.0253402
0.2.30.1.2.4.3	Pediococcus	0.0040065	0.0257258
0.2.2.1.2.25.6	Micrococcus	0.0053285	0.0325040
0.2.30.1.2.4.1	Lactobacillus	0.0074849	0.0428767
0.2.5.4.1.2.11	Chryseobacterium	0.0077319	0.0428767
0.2.30.2.1.1.4	Clostridium_sensu_stricto	0.0083236	0.0441512
0.2.30.1.1.12.14	Sporosarcina	0.0087437	0.0444474
0.2.30.1.2.4.4	unclassified	0.0102387	0.0499647
