# Supplementary figures and images for: Naturally Fermented Milk From Northern Senegal: Bacterial Community Composition and Probiotic Enrichment With Lactobacillus rhamnosus
Source: Front Microbiol. 2018 Sep 21;9:2218. doi: 10.3389/fmicb.2018.02218 (PMC6160551; doi:10.3389/fmicb.2018.02218)

Heat map of microbiota compositions of lait caillé starters tablets (10) and lahal biofilms (20).

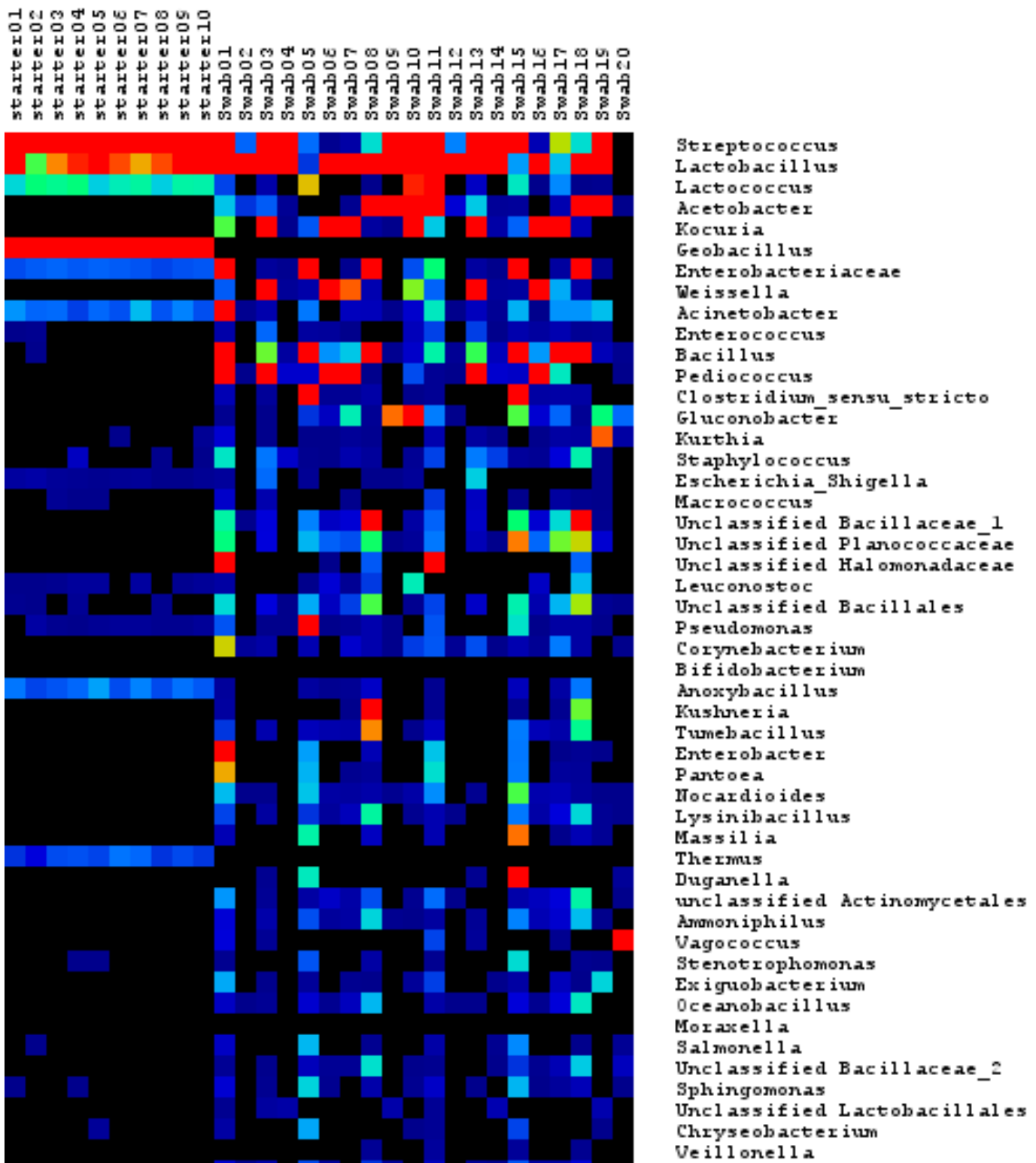

Supplement: FILE S1 — Raw data sheet sequence reads. The data sheet contains all the sequence reads for all taxons up to the genus level identified in the naturally fermented milk samples of all six communities, collected starter cultures, commercial yogurts, and lahal biofilms. [file Data_Sheet_1.ZIP › Supplemental file S5.pdf]
